# Low-Complexity Wideband Interference Mitigation for UWB ToA Estimation

**DOI:** 10.3390/s23135806

**Published:** 2023-06-21

**Authors:** Stefan Hechenberger, Stefan Tertinek, Holger Arthaber

**Affiliations:** 1 Institute of Electrodynamics, Microwave and Circuit Engineering, TU Wien, 1040 Vienna, Austria; stefan.hechenberger@tuwien.ac.at; 2 Christian Doppler Laboratory for Location-Aware Electronic Systems, 8010 Graz, Austria; 3 NXP Semiconductors, 8101 Gratkorn, Austria

**Keywords:** UWB, localization, interference, time of arrival, angle of arrival, LCMV, low-complexity, array processing

## Abstract

Reliable time of arrival (ToA) estimation in dense multipath (DM) environments is a difficult task, especially when strong interference is present. The increasing number of multiple services in a shared spectrum comes with the demand for interference mitigation techniques. Multiple receiver elements, even in low-energy devices, allow for interference mitigation by processing coherent signals, but computational complexity has to be kept at a minimum. We propose a low-complexity, linearly constrained minimum variance (LCMV) interference mitigation approach in combination with a detection-based ToA estimator. The performance of the method within a realistic multipath and interference environment is evaluated based on measurements and simulations. A statistical analysis of the ToA estimation error is provided in terms of the mean absolute error (MAE), and the results are compared to those of a band-stop filter-based interference blocking approach. While the focus is on receivers with only two elements, an extension to multiple elements is discussed as well. Results show that the influence of strong interference can be drastically reduced, even when the interference bandwidth exceeds 60% of the signal bandwidth. Moreover, the algorithm is robust to uncertainties in the angle of arrival (AoA) of the desired signal. Based on these results, the proposed mitigation method is well suited when the interference bandwidth is large and when computational power is a critical resource.

## 1. Introduction

The location-awareness of electronic devices has become an integral element for a variety of applications. Secure access-granting is one example where reliable, accurate range estimation is of major importance. Although the superior time resolution of ultra-wideband (UWB) signals allows for high-accuracy time of arrival (ToA) estimation in line of sight (LoS) scenarios, reliable estimation is still a demanding task in harsh environments including dense multipath (DM) and interference. While ToA estimation in DM scenarios has been the subject of extensive research, e.g., [1,2,3,4], discussion of the problem of interference mitigation within the UWB ToA estimation framework is rather sparse in existing literature. With a rising number of services sharing the same spectrum, this becomes an increasingly important issue. Frequency bands at 5.945–6.425 GHz in Europe [5] and at 5.925–7.125 GHz in the USA [6] are now available for unlicensed transmission, and overlap to a large extent with channel 5 and 6 of the high-repetition pulse physical layer (HRP PHY) of the IEEE 802.15.4 standard [7], centered at 6.490 GHz and 6.989 GHz. Wi-Fi will arguably be the most prominent service within these frequency bands in the near future. With a maximum channel bandwidth of 160 MHz in Wi-Fi 6E and 320 MHz in Wi-Fi 7 [8], a single Wi-Fi channel occupies up to 64% of the UWB signal bandwidth of 500 MHz.

Low-energy devices, such as a key fob in an access granting system, are strictly limited in their power consumption to ensure a long life-cycle. State-of-the art devices with one receiver use band-stop filters (BSF) to mitigate the influence of interference signals. However, a large bandwidth of the interference signal strongly limits the performance of BSFs. More sophisticated signal processing techniques are enabled by multiple coherent receivers, which are recently becoming available even in low-energy UWB devices. When computational power is at a premium, optimal estimators, such as the maximum likelihood (ML) estimator, can be implemented, incorporating statistical models of the DM and the interference. For low-energy devices, the computational complexity of these estimators is prohibitive in general. Furthermore, the second-order statistics of the DM and the interference signal are often unknown in practice.

We propose a combined interference mitigation and ToA estimation method of low complexity. The ToA estimation is performed by an LoS detection algorithm which performs well under good signal-to-interference-plus-noise ratio (SINR) conditions, even in non-line of sight (NLoS) scenarios. In order to obtain the required SINR, we implement a linearly constrained minimum variance (LCMV) processor prior to the ToA estimator. It is shown that a low-dimensional implementation of the LCMV processor, combined with the detection-based ToA estimation, obtains good results in the presence of wideband interference and is indeed of low complexity. In the presentation of the results, we will focus on the case of a 2×1 linear array, but the discussion will be extended to the higher-dimensional case.

### 1.1. Related Work and Contribution

The impact of interference on UWB systems has been the subject of research, but the vast majority deals with UWB communication systems under the assumption of narrowband interference. Performance analyses of UWB communication systems in the presence of narrowband interference for different receiver types are given in [9,10,11,12], and narrowband interference mitigation schemes are presented in [13,14,15,16]. The performance of UWB ToA estimation is analyzed in the presence of narrowband and multi-user interference [17,18] and wideband interference [19]. The mitigation of wideband interference in UWB ToA systems has not been investigated thoroughly. This might be explained by the fact that its practical relevance is only recently emerging due to the availability of the 6 GHz-band for unlicensed transmission. A general approach to wideband interference mitigation can be found in the wideband beamforming literature, e.g., [20], including the LCMV processor.

The general solution to the LCMV problem was derived in [21]. In [21,22], algorithms for adaptive calculation of the optimal solution were developed, where the pre-steering of the receiver array was assumed. In [23], this was generalized to the case of an arbitrary angle of arrival (AoA) without pre-steering, and the most efficient low-rank approximation for this case was found. The processor obtained for a simple set of point constraints, as in [21,22,23], is generally quite sensitive to deviations of the AoA and other parameters. In [24,25,26], the original approach was modified in various ways in order to improve the robustness with regard to parameter deviations. In this work, we assume no pre-steering of the receiver array [23], and we implement the robust method based on a probabilistic approach in [25]. Both choices are motivated by the requirement of low complexity. A good overview of additional work is given in [20].

In the literature referred to above, the analysis of the LCMV processor is limited to a few (mostly two) interference sources with independent signals and significantly separated locations. In this work, the interference signal is assumed to originate from a single source and to arrive at the UWB receiver after propagation over a DM. This means that the interference signal impinges from many different directions, including both spatially correlated and uncorrelated components. The interference mitigation capability of the LCMV processor in such a scenario has not been investigated yet. Furthermore, the performance analysis in the LCMV literature is typically carried out for a large number of antenna elements and a large filter-length. In this work, we show that a low-dimensional implementation of the LCMV processor is able to significantly reduce the performance degradation of the ToA estimation due to wideband interference. Although the LCMV approach has been used in the context of ToA estimation and localization [27,28,29], it was only used with the purpose of self-interference mitigation. Research on the mitigation of wideband interference based on LCMV processing in UWB ToA estimation is not known to the authors.

Given the discussion above, the key contributions of this paper are briefly listed as follows:

- Adoption of the presented signal model to the LCMV processor
- Presentation of a low-dimensional implementation of the LCMV processor in combination with a detection-based ToA estimator
- Virtual array measurements of a UWB indoor channel
- Statistical evaluation of ToA estimation error based on measurements and simulations

### 1.2. Paper Outline

In Section 2, we discuss the signal model of multiple receivers in the presence of DM and interference. The combined interference mitigation and ToA estimation method is presented in Section 3. The indoor channel measurement campaign is described in Section 4, and the results are presented in Section 5.

## 2. Signal Model

### 2.1. Receive Signal

A UWB pulse s(t) is transmitted from a single transmit antenna to *K* receive antennas. Considering propagation over a multipath channel and perturbation by additive interference and noise, the equivalent complex-valued baseband signal at the *k*th receive antenna after the matched filter is

(1) $$\begin{array}{c} r^{\left(k\right)}\left(t\right)=g^{\left(k\right)}\left(t\right)+g\left(t\right)*\nu^{\left(k\right)}\left(t\right)+p^{\left(k\right)}\left(t\right)\;, \end{array}$$

where g(t) is the autocorrelation of the pulse, $\nu^{\left(k\right)}\left(t\right)$ is the dense multipath component (DMC), ∗ denotes the convolution operator, and $p^{\left(k\right)}\left(t\right)$ is the perturbation signal. The deterministic signal part $g^{\left(k\right)}\left(t\right)$ received via the LoS is

(2) $$\begin{array}{c} g^{\left(k\right)}\left(t\right)=\alpha^{\left(k\right)}\;g\left(t-\tau^{\left(k\right)}\right)\;e^{j\varphi_{0}}\;, \end{array}$$

where $\alpha^{\left(k\right)}$ and $\tau^{\left(k\right)}$ are the attenuation and delay introduced by the distance between the transmitter and the *k*th receive antenna, and $\varphi_{0}∼U\left(0,2\pi \right)$ is a uniformly distributed carrier phase offset, which is equal for all receive elements due to a shared phase reference. The DMC $\nu^{\left(k\right)}\left(t\right)$ is a complex random process with no further specification, and the perturbation signal $p^{\left(k\right)}\left(t\right)$ includes interference and noise and is specified in Section 2.2. Let $q^{\left(0\right)}$ be a reference point in the vicinity of the receive antenna locations $q^{\left(k\right)}$ ($q^{\left(0\right)}$ is typically chosen to be the geometrical center of the receiver array) such that

(3) $$\begin{array}{c} \alpha^{\left(0\right)}\approx \alpha^{\left(k\right)}\;\forall \;k\in \left\{1,2,\ldots ,K\right\}\;. \end{array}$$

This means that the deterministic signal part $g^{\left(k\right)}\left(t\right)$, given by Equation (2), is approximately

(4) $$\begin{array}{c} g^{\left(k\right)}\left(t\right)\approx F^{-1}\left\{G^{\left(0\right)}\left(\omega \right)\;e^{j\Delta \varphi^{\left(k\right)}\left(\omega ,\theta \right)}\right\}\;, \end{array}$$

where $F^{-1}$ is the inverse Fourier-transform, and $G^{\left(0\right)}\left(\omega \right)$ is the Fourier-transform of $g^{\left(0\right)}\left(t\right)$. The phase difference $\Delta \varphi^{\left(k\right)}$ between $q^{\left(0\right)}$ and $q^{\left(k\right)}$ for a signal with an AoA of θ∈[0,2π) is a function of $q^{\left(0\right)}$, $q^{\left(k\right)}$, θ, and ω ( extension to the three-dimensional case is straightforward by adding an elevation angle to the model). The dependency on $q^{\left(0\right)}$ and $q^{\left(k\right)}$ in Equation (4) is not stated explicitly. For a given θ, the desired signal part $g^{\left(k\right)}\left(t\right)$ at the *k*th receive element is therefore solely expressed through $g^{\left(0\right)}\left(t\right)$. Note that Equation (4) corresponds to a shift of $g^{\left(0\right)}\left(t\right)$ in the time domain. Frequency domain representation will be used in the method described in Section 3 and is therefore chosen here. It is further noted that this is different from the narrowband assumption, due to the large bandwidth of the transmit signal s(t).

### 2.2. Interference

The perturbation signal in Equation (1) is given by

(5) $$\begin{array}{c} p^{\left(k\right)}\left(t\right)=u^{\left(k\right)}\left(t\right)+z^{\left(k\right)}\left(t\right)\;, \end{array}$$

where $u^{\left(k\right)}$ and $z^{\left(k\right)}$ denote the interference and noise, respectively. While the zero-mean, white Gaussian noise is i.i.d. for all *k*, the interference signal is correlated across the receive elements, which is used by the method described in Section 3 to reduce the interference power. The interference signal $u_{I}\left(t\right)$ is transmitted by a single source and received by the *k*th element via the interference channel $h_{I}^{\left(k\right)}\left(t\right)$. The received interference signal at antenna *k* is thus

(6) $$\begin{array}{c} u^{\left(k\right)}\left(t\right)=u_{I}\left(t\right)*h_{I}^{\left(k\right)}\left(t\right)\;, \end{array}$$

where $u_{I}\left(t\right)$ is a wide-sense stationary (WSS) signal, effectively bandlimited to $B_{I}$. The interference channel $h_{I}^{\left(k\right)}\left(t\right)$ includes the direct path and multipath components and is described by a random process. It is unknown to the receiver, and no knowledge about its statistical properties is assumed. In a beamforming framework, this can be interpreted as follows. Due to $h_{I}^{\left(k\right)}\left(t\right)$, the signal $u_{I}\left(t\right)$ impinges upon the receiver array from multiple angles with different delays resulting in $u^{\left(k\right)}\left(t\right)$, but the number of signal components and the angles at which they arrive at the receiver are unknown.

### 2.3. Time-Discretization

The receive signal is sampled at frequency $f_{s}=1/T_{s}$, where $T_{s}$ is the sampling time. In the following, boldface vector notation will be used to represent the sampled version of a signal, e.g., $s=[s_{0},s_{1},\ldots ,s_{N-1}]$, with $s_{n}=s\left(nT_{s}\right)$, *N* being the number of samples, and $T=NT_{s}$ the observation duration. The sampled version of the baseband signal is then

(7) $$\begin{array}{c} r^{\left(k\right)}=g^{\left(k\right)}+v^{\left(k\right)}+p^{\left(k\right)}\;, \end{array}$$

where $g^{\left(k\right)}$ is the sampled version of the delayed pulse, given in Equation (2), and $v^{\left(k\right)}$ is the sampled version of the pulse convolved with the DMC in Equation (1). The perturbation vector is given by

(8) $$\begin{array}{c} p^{\left(k\right)}=u^{\left(k\right)}+z^{\left(k\right)}\;, \end{array}$$

where $u^{\left(k\right)}$ and $z^{\left(k\right)}$ are the sampled versions of the received interference and noise.

## 3. LCMV Processing and ToA Estimation

**Figure 1:**
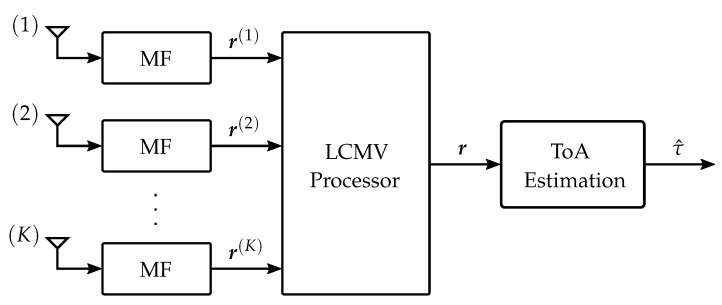
Block diagram of the receiver processing and ToA estimation. Each of the *K* receive antennas is followed by an MF. The *K* sampled receive signals $r^{\left(k\right)}$ after the MF are combined by the LCMV processor into a single vector r. Based on the resulting vector r, the ToA is estimated.

This section describes the combined approach to the interference mitigation and ToA estimation. Figure 1 shows the overall structure of the receiver processing. The *K* receive signals $r^{\left(k\right)}$, given by Equation (7), are combined by the LCMV processing in order to obtain a single signal r with an increased SINR. Based on this result, the ToA $\tau^{\left(0\right)}$, which corresponds to the distance between the transmitter location and the reference point $q^{\left(0\right)}$, is estimated by a detection-based estimator.

### 3.1. Problem Formulation

**Figure 2:**
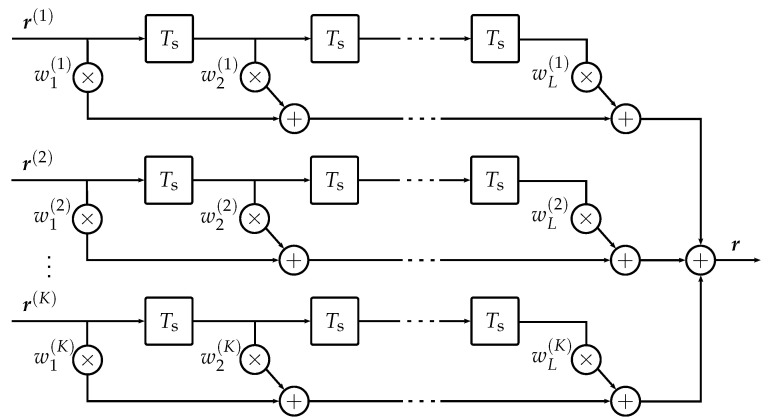
Filter-and-sum structure of the LCMV processor (revised from [22]). Each of the *K* receive vectors $r^{\left(k\right)}$ is passed through a finite impulse response filter of length *L* with filter coefficients $w_{l}^{\left(k\right)}$ and a tap-delay of $T_{s}$. The resulting vectors are summed up to obtain the single receive vector r.

We start with a brief overview of the LCMV processor [21,22] and emphasize the motivation for choosing this approach within the present framework. Figure 2 shows the filter-and-sum structure of the LCMV processor. The vectors $r^{\left(k\right)}$ are the *K* sampled receive signals after each matched filter (MF) (cf. Figure 1). They are the input for *K* finite impulse response filters of length *L*, and the resulting output vectors are combined to obtain a single vector r. The KL complex-valued filter coefficients $w_{l}^{\left(k\right)}$ shall be chosen such that the following two goals are achieved:

(i) The resulting interference power is minimized.
(ii) The envelope of the desired signal is preserved.

The motivation for using the LCMV processing in combination with the detection-based ToA estimation is the realization of goals (i) and (ii) by means of low complexity. It has been shown, in [19], that the estimation error due to multipath propagation produced by the detection-based ToA estimator tends to be small under good SINR conditions, which shall be ensured by the realization of (i). The local error in the vicinity of the LoS due to pulse distortion effects is minimized by the realization of (ii). A formal description of this problem follows.

Consider the input matrix with delay index $m\in \{0,1,\ldots ,N_{}-L\}$, in structural analogy to Figure 2,

(9) $$X_{m}=\left[\begin{array}{cccc} r_{m}^{\left(1\right)} & r_{m+1}^{\left(1\right)} & \cdots & r_{m+L-1}^{\left(1\right)}\\ r_{m}^{\left(2\right)} & r_{m+1}^{\left(2\right)} & \cdots & r_{m+L-1}^{\left(2\right)}\\ \vdots & \vdots & \ddots & \vdots \\ r_{m}^{\left(K\right)} & r_{m+1}^{\left(K\right)} & \cdots & r_{m+L-1}^{\left(K\right)} \end{array}\right]\;,$$

and the stacked input vector

(10) $$\begin{array}{c} x_{m}=\mathrm{vec}\left(X_{m}\right)\;, \end{array}$$

where the vectorization operator vec(·) stacks the columns of a matrix on top of each other. If the weighting matrix and the stacked weighting vector are constructed in the same fashion, i.e.,

(11) $$W=\left[\begin{array}{cccc} w_{1}^{\left(1\right)} & w_{2}^{\left(1\right)} & \cdots & w_{L}^{\left(1\right)}\\ w_{1}^{\left(2\right)} & w_{2}^{\left(2\right)} & \cdots & w_{L}^{\left(2\right)}\\ \vdots & \vdots & \ddots & \vdots \\ w_{1}^{\left(K\right)} & w_{2}^{\left(K\right)} & \cdots & w_{L}^{\left(K\right)} \end{array}\right]\;,$$

and

(12) $$\begin{array}{c} w=\mathrm{vec}(W)\;, \end{array}$$

then the *m*th element of the output vector r is given by the inner product of the weighting and input vectors

(13) $$\begin{array}{c} r_{m}=w^{H}x_{m}\;. \end{array}$$

By inserting Equation (7) into Equation (9) and Equation (9) into Equation (10), the stacked input vector $x_{m}$ can be rewritten as

(14) $$\begin{array}{c} x_{m}=g_{m}+v_{m}+p_{m}\;, \end{array}$$

where $g_{m}$, $v_{m}$, and $p_{m}$ are the stacked pulse vector, stacked DMC vector, and stacked perturbation vector. Equation (13) is then rewritten as

(15) $$\begin{array}{c} r_{m}=w^{H}g_{m}+w^{H}v_{m}+w^{H}p_{m}\;. \end{array}$$

Under the assumption of statistical independence and zero-mean property of all three components, the covariance matrix of $x_{m}$ is

(16) $$\begin{array}{c} R_{x_{m}x_{m}}=R_{g_{m}g_{m}}+R_{v_{m}v_{m}}+R_{p_{m}p_{m}}\;, \end{array}$$

where

(17) $$R_{g_{m}g_{m}}=E_{}\left\{g_{m}g_{m}^{H}\right\}\;,$$

(18) $$R_{v_{m}v_{m}}=E_{}\left\{v_{m}v_{m}^{H}\right\}\;,$$

and

(19) $$R_{p_{m}p_{m}}=E_{}\left\{p_{m}p_{m}^{H}\right\}\;$$

are the pulse covariance matrix, DMC covariance matrix, and perturbation covariance matrix, respectively. Jointly realizing goals (i) and (ii) can now be formulated as a constrained minimization problem:

(20a) $$\begin{array}{cccc} & \min_{w} & & \;w^{H}R_{p_{m}p_{m}}w \end{array}$$

(20b) $$\begin{array}{cccc} & \mathrm{subject}\;\mathrm{to} & & \;C^{H}w=f\;, \end{array}$$

where $C\in C^{KL\times J}$ is the constraint matrix, and $f\in C^{J}$ is the response vector. That is, the mean power $w^{H}R_{p_{m}p_{m}}w$ of the *m*th element of the stacked perturbation vector $p_{m}$ is minimized with regard to w, while the latter has to fulfill the constraint of Equation (20b). Equations (20a) and (20b) are a quadratic program with the well known solution found by the method of Lagrange multipliers [21],

(21) $$w_{\mathrm{opt}}=R_{p_{m}p_{m}}^{-1}C{\left(C^{H}R_{p_{m}p_{m}}^{-1}C\right)}^{-1}f\;.$$

Note that $w_{\mathrm{opt}}$ does not depend on the delay index *m*. It will be shown in Section 3.3 that, for a stationary perturbation signal, the covariance matrix $R_{x_{m}x_{m}}$ and, as a consequence, the optimal weighting vector $w_{\mathrm{opt}}$ do not depend on *m*.

For any given AoA of the desired signal component, the constraint matrix C and the response vector f can be pre-calculated. The design of these constraints is discussed in Section 3.2. The perturbation covariance matrix $R_{p_{m}p_{m}}$ is unknown a priori and has to be estimated, as described in Section 3.3.

### 3.2. Constraint Equation

We will now discuss the design of the constraints and the choice of optimal parameters with regard to the ToA estimation. The constraint equation is responsible for the realization of goal (ii), which is an undistorted envelope of the desired signal $g^{\left(k\right)}$. In the frequency domain, this is achieved by a constant gain and linear phase spectrum. Pre-steering of the receiver array ( the virtual look-direction of the array could be steered to a desired AoA by placing wideband filters in front of the LCMV processor), as assumed in [21], is not employed because it would come at the cost of computational expense. Alternatively, the steering can be incorporated into the constraint matrix C [20]. If a signal with an AoA of θ and bandwidth *B* excites the receiver structure given in Figure 2, C is constructed according to

(22) $$C=\left[c_{1},c_{2},\ldots ,c_{J}\right]\;,$$

where the *j*th column is

(23) $$c_{j}=\mathrm{vec}\left(\left[\begin{array}{cccc} e^{j\left(\Delta \varphi^{\left(1\right)}\left(\omega_{j},\theta \right)\right)} & e^{j\left(\Delta \varphi^{\left(1\right)}\left(\omega_{j},\theta \right)+\omega_{j}T_{s}\right)} & \cdots & e^{j\left(\Delta \varphi^{\left(1\right)}\left(\omega_{j},\theta \right)+\left(L-1\right)\omega_{j}T_{s}\right)}\\ e^{j\left(\Delta \varphi^{\left(2\right)}\left(\omega_{j},\theta \right)\right)} & e^{j\left(\Delta \varphi^{\left(2\right)}\left(\omega_{j},\theta \right)+\omega_{j}T_{s}\right)} & \cdots & e^{j\left(\Delta \varphi^{\left(2\right)}\left(\omega_{j},\theta \right)+\left(L-1\right)\omega_{j}T_{s}\right)}\\ \vdots & \vdots & \ddots & \vdots \\ e^{j\left(\Delta \varphi^{\left(K\right)}\left(\omega_{j},\theta \right)\right)} & e^{j\left(\Delta \varphi^{\left(K\right)}\left(\omega_{j},\theta \right)+\omega_{j}T_{s}\right)} & \cdots & e^{j\left(\Delta \varphi^{\left(K\right)}\left(\omega_{j},\theta \right)+\left(L-1\right)\omega_{j}T_{s}\right)} \end{array}\right]\right)\;,$$

and the frequency points $\omega_{j}$ are evenly spaced within [−πB,πB]. The phase difference $\Delta \varphi^{\left(k\right)}\left(\omega_{j},\theta \right)$ is determined by the array structure and the AoA, and is the same as in Equation (4). Equation (23) shows that each column of C corresponds to the excitation of the combined array and filter-and-sum structure by a tone of frequency $\omega_{j}$ with an AoA of θ. The unit gain and linear phase requirement for the desired signal results in a response vector f with elements

(24) $$f_{j}=e^{j\omega_{j}\tau_{f}}\;,$$

where $\tau_{f}$ is the resulting filter delay. The dimensions of C given by KL and *J* determine the degrees of freedom available to control the filter response. For J<KL and C of full rank, the degrees of freedom are equal to KL−J. If *K* and *L* are fixed, the problem is to find the best value for *J*. If the chosen *J* is too small, then not enough constraints are imposed on the response in AoA-direction, and the desired signal is distorted. This results in an increased error in the ToA estimation around in the vicinity of the LoS. If the chosen *J* is too large, the degrees of freedom are too low for sufficient interference suppression. This results in an increase in errors introduced by interference and multipath components. Furthermore, the conditioning of C depends on θ, and thus, C is not of full rank in general. Consequently, the effective degrees of freedom and the relation to the number of constraints *J* are dependent on θ as well. This has to be considered in the search for the optimal values of these parameters. An optimality criterion in terms of ToA estimation is, for example, the mean squared error (MAE) or the variance of the estimation error in the vicinity of the LoS. As there is no analytical solution apparent to such a criterion, it has to be found empirically. Results are presented in Section 5.

### 3.3. Estimation of the Perturbation Covariance Matrix

It was established in Section 2.2 that the transmitted interference signal $u_{I}\left(t\right)$ is WSS. If the transmission duration of $u_{I}\left(t\right)$ is at least as long as the observation duration *T* and the interference channel $h_{I}^{\left(k\right)}$ is assumed to be static within *T*, then the stacked perturbation vector $p_{m}$ is WSS as well. This means that the perturbation covariance matrix $R_{p_{m}p_{m}}$ is independent of the delay index *m*, i.e.,

(25) $$R_{p_{m}p_{m}}=R_{p_{n}p_{n}}\;\forall \;m,n\in \left\{0,1,\ldots ,N-L\right\}\;.$$

**Figure 3:**
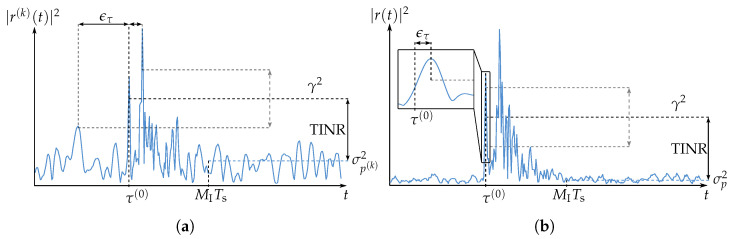
An example of the estimated channel impulse response in a NLOS scenario is shown (**a**) before and (**b**) after the LCMV processing.

However, $R_{p_{m}p_{m}}$ is unknown and has to be estimated. For an observation time *T* larger than the maximal delay of the DMC term $v_{m}$, this can be achieved by the sample method

(26) $${\hat{R}}_{p_{m}p_{m}}=\frac{1}{M_{R}}\sum_{n=M_{I}}^{M_{I}+M_{R}-1}x_{n}x_{n}^{H}\;,$$

where $M_{I}$ is an offset index and $M_{R}$ is the number of sample vectors used for the estimation. The offset index $M_{I}$ is chosen to be large enough so that no multipath components, but only interference and noise, are assumed to be present in $x_{m}$ (cf. Figure 3). Finally, $R_{p_{m}p_{m}}$ in Equation (21) is replaced by ${\hat{R}}_{p_{m}p_{m}}$.

It is emphasized that all quantities involved in the computation of the optimal filter coefficients $w_{\mathrm{opt}}$ in Equation (21), with the exception of ${\hat{R}}_{p_{m}p_{m}}$, can be calculated offline for all possible θ because they do not depend on the data vector $x_{m}$. This is an important fact if computational power is a critical resource.

### 3.4. Spatial Flattening

When the constraint matrix C is calculated with regard to a certain AoA θ, the resulting filter is, in general, quite sensitive to deviations in θ. If θ has to be estimated and is subject to errors, this can result in substantial performance degradation. One approach to mitigate this problem is to include additional derivative constraints in the constraint Equation (20b) [24]. In the case of a linear receiver array, *J* is increased by a factor of two for first-order derivative constraints, and by a factor of three for second-order derivative constraints. Even though C can be calculated offline, it is also involved in the computation of the optimal weight coefficients in Equation (21). Thus, an increased dimensionality of C results in an increase in the calculations to be carried out adaptively. For this reason, a probabilistic spatial flattening approach, proposed in [25], is chosen. Let us consider that θ is a random parameter with a probability density function (pdf) p(θ). Then, the mean constraint matrix $\bar{C}$ is given by

(27) $$\bar{C}=\int_{\theta =0}^{2\pi }C\left(\theta \right)p\left(\theta \right)\;d\theta \;,$$

and the constraint matrix C in Equation (21) is replaced by Equation (27). It is evident that the dimensionality of the constraint matrix, and as a result, the computational complexity, does not increase. In a practical implementation, θ takes on discrete values, and the integral is replaced by a sum.

Note that a spatial flattening method comes at the cost of reduced array gain. If p(θ) is rather flat, then the LCMV processing becomes more robust to deviations in θ, but the gain in the direction of the true θ is reduced. For a narrow p(θ), this relation is inverted.

### 3.5. ToA Estimation

We will now discuss the estimation of the ToA $\tau^{\left(0\right)}$. It is shown in Figure 1 that the input for the ToA estimation is the signal r obtained by the LCMV processor. As for the interference mitigation, low computational complexity is crucial for the ToA estimation. For this purpose, a detection-based estimator is implemented, which searches for the first peak of r exceeding a certain threshold γ. This is written as

(28) $$\begin{array}{cc} {\hat{m}}^{\left(0\right)}=\;\; & \min\;\;\;\;\;\;m\\ \; & \mathrm{subject}\;\mathrm{to}\;|r_{m}|>\gamma ,\\ \; & \;\;\;\;\;\;|r_{m-1}\left|\leq \right|r_{m}\left|\geq \right|r_{m+1}| \end{array}\;,$$

where $r_{m}$ is given in Equation (13), and ${\hat{m}}^{\left(0\right)}$ is the estimated discrete-time index corresponding to the ToA $\tau^{\left(0\right)}$. The threshold γ is chosen with regard to the mean power $\sigma_{p}^{2}$ of the residual perturbation vector p, of which the elements are given by the last term in Equation (15). This is an unknown parameter and is estimated by

(29) $${\hat{\sigma }}_{p}^{2}=\frac{1}{M_{R}}\;\sum_{m=M_{I}}^{M_{I}+M_{R}-1}{\left|r_{m}\right|}^{2}\;,$$

where, again, $M_{I}$ is chosen to be large enough that only interference and noise are assumed to be present in $r_{m}$, as indicated in Figure 3. The threshold γ is then defined via the threshold-to-interference-plus-noise ratio

(30) $$\mathrm{TINR}=10\log_{10}\left(\frac{\gamma^{2}}{\sigma_{p}^{2}}\right)\;.$$

After the index ${\hat{m}}^{\left(0\right)}$ is found, peak interpolation is performed by a parabolic function f(·), and the ToA is estimated by

(31) $${\hat{\tau }}^{\left(0\right)}=\underset{\tau }{\arg\;\max}\;\;f(\tau ,|r_{{\hat{m}}^{\left(0\right)}-1}\left|,\right|r_{{\hat{m}}^{\left(0\right)}}\left|,\right|r_{{\hat{m}}^{\left(0\right)}+1}\left|\right)\;.$$

It has been shown, in [19], that such an estimator performs well in multipath environments when operated at a sufficiently high SINR. Figure 3 schematically represents the combination of the LCMV processing and the ToA estimation. In Figure 3a, an example of a channel estimate at a single receive element is shown, where the estimation error is given by

(32) $$\epsilon_{\tau }={\hat{\tau }}^{\left(0\right)}-\tau^{\left(0\right)}\;.$$

If the SINR is low, then $\epsilon_{\tau }$ is outlier-driven (this has also been shown for the ML estimator in [30]). This means that $\epsilon_{\tau }$ is introduced by detecting an interference peak or a multipath component instead of the peak at the true ToA $\tau^{\left(0\right)}$. Errors of this type are also called global errors. The goal of the LCMV processing is to obtain a combined channel estimate with an SINR, such that the $\epsilon_{\tau }$ is not governed by global errors. This is shown in Figure 3b. Furthermore, local errors, introduced by distortion of the pulse at $\tau^{\left(0\right)}$, are minimized by imposing unit gain and linear phase constraints on the filtering of the desired signal. It is shown in Section 5 that there is a trade-off between these two types of errors in parameterizing the LCMV processing.

## 4. Measurement Setup

**Figure 4:**
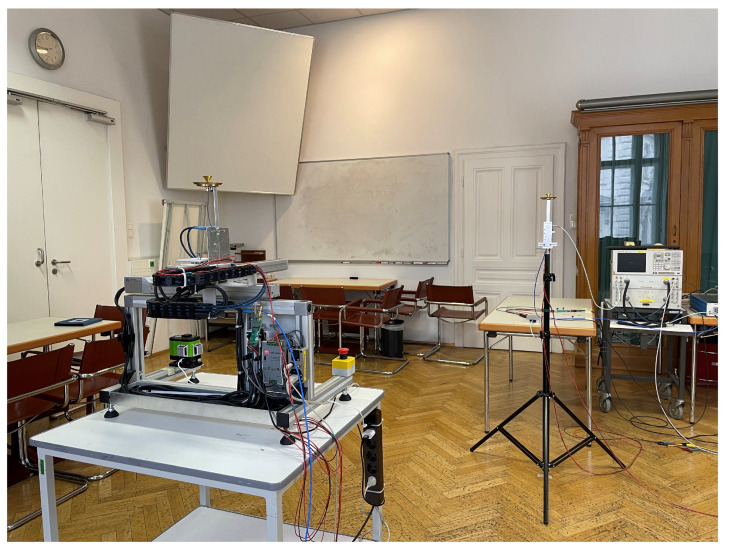
Channel measurement environment: seminar room at TU Wien. Specular components, e.g., walls, white board, and cabinet with glass front, as well as diffuse components, e.g., tables, chairs, and measurement equipment, are part of the interior.

In order to evaluate the method described in Section 3 in a realistic environment, channel measurements were performed in a seminar room at the TU Wien, shown in Figure 4. The room measures 7.8 m×7.8 m×3.8 m, and the interior includes tables, chairs, measurement equipment, three large wooden cabinets with glass front, a shelf, and a metallic white board, all placed at the sides of the room to create enough space for the antenna placement. The right antenna in Figure 4 is mounted on a static tripod at a height of 1.55 m and is connected to the vector network analyzer (vna) via coaxial cable. The left antenna is mounted at the same height on a two-dimensional, horizontal positioner for fine antenna placement with sub-millimeter accuracy, and the positioner is placed on a wheeled table for coarse placement in the room. The antenna is connected to a coaxial cable that winds down along the two axes using a cable carrier to guarantee a defined bending of the cables. From there, through a fixed connector, another coaxial cable is connected to the second port of the vna. While the latter has to be slightly moved for each table position, the cable along the axes experiences a different bending for each position of the axes. Although measurements showed good phase stability of both cables, it should be mentioned that this might have a small impact on the phase accuracy of the measurements, as calibration between the two antenna ports is carried out for a single position of the table and axes. Aside from this fact, the measurements are phase coherent, which is a crucial requirement for the investigated method.

**Figure 5:**
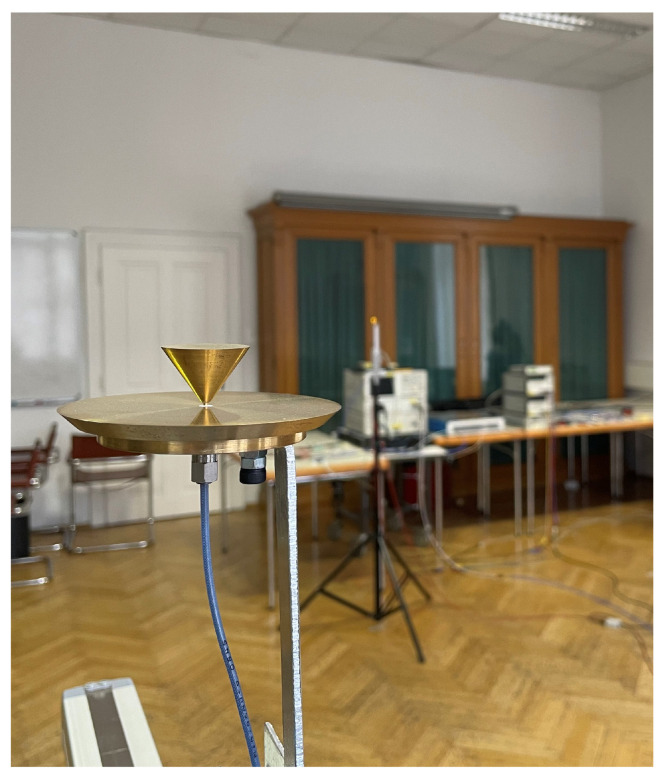
CMP mounted on the positioner. In the back, one of the wooden cabinets with glass fronts is seen.

The same antenna, shown in Figure 5, is used at the transmitter and receiver sides. It is a redesign of the conical monopole antenna (CMP) introduced in [31]. The choice of the CMP is motivated by the requirement for a constant gain within the frequency span of interest and by radial symmetry of the antenna. Furthermore, the measurement results are comparable to previous measurements with the purpose of ToA estimation, as in, e.g., [32]. With calibration between the two antenna ports, the CMPs are considered part of the channel.

Measurements are performed by the vna over a bandwidth of 998.4 MHz at center frequency $f_{c}=6.49\;G\mathrm{Hz}$. The number of frequency points is 1016, and the IF-bandwidth is 3 kHz. This results in a time-domain signal duration rounded to T=1016 ns and a sample time $T_{s}=1\;ns$. The parameters are chosen to coincide with the parameters of the HRP UWB physical layer in the IEEE 802.15.4 standard [7] for the longest ranging sequence at channel 5.

A total of 8000 measurements were performed in a rectangular area of 3.4 m×2.7 m. Per table position, the axes’ positions are on a 10×10 grid with equidistant spacing of half a wavelength at center frequency $f_{c}$. It is noted that the characterization of the channel in the form of a power delay profile (PDP) does not require 8000 measurements. However, another objective is to form virtual antenna arrays for the statistical evaluation of the method described in Section 3. In order to form arrays of variable dimension and orientation and still have enough data points to obtain reliable statistics, a large number of measurement locations was chosen. Virtual antenna arrays are only formed within the locations of one table position, to guarantee high accuracy spacing between the elements.

**Figure 6:**
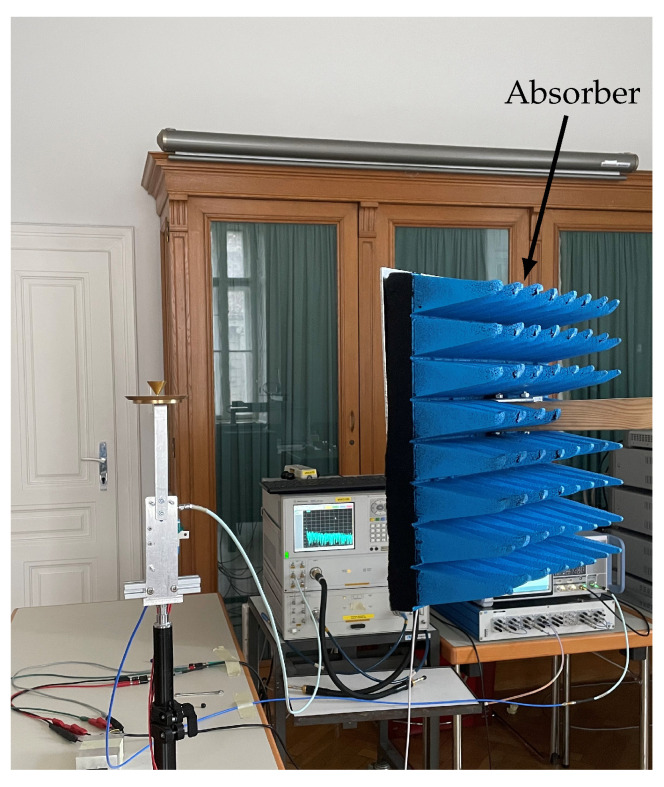
Absorber placed in front of the antenna for NLOS conditions.

Especially problematic for ToA estimation within multipath environments are NLOS scenarios, where the path between transmitter and receiver is obstructed. For this purpose, an absorber mounted on a tripod was placed between the antennas, as shown in Figure 6.

**Figure 7:**
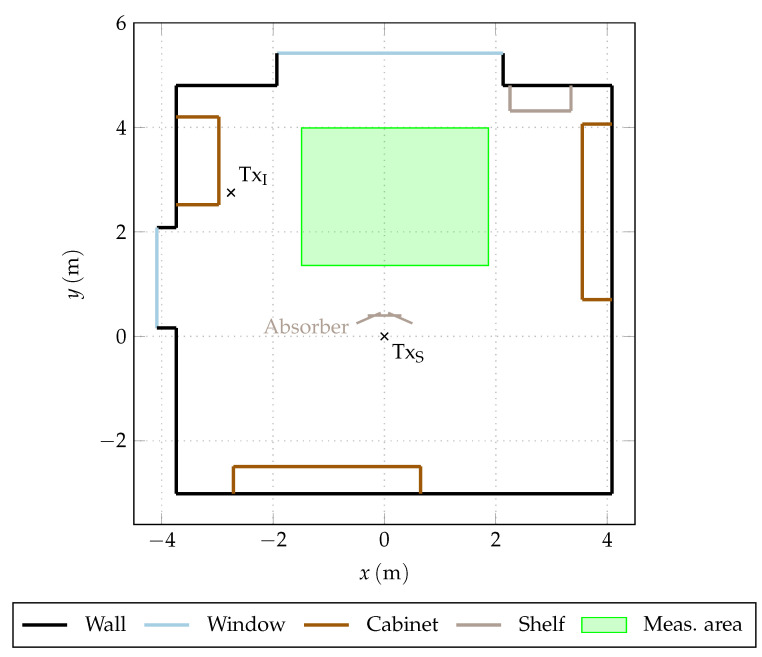
Floor plan of the seminar room. Large objects in the interior are shown in different colors according to their material. The antenna positions of both the UWB source (${\mathrm{Tx}}_{S}$) and the interference source (${\mathrm{Tx}}_{I}$) are indicated by the crosses, and the green region is the area of the receiver placement.

The measurements of the interference channel are performed in the same fashion, but with the static antenna, which in this case represents the interference source location, placed in a different area of the room. The path between the transmitter and receiver of the interference channel was always free of obstructions. The exact locations of transmitter and receiver placements are shown in Figure 7.

## 5. Results

In this section, we evaluate the method proposed in Section 3 within the multipath environment described in Section 4. The performance of the method in terms of the ToA estimation error depends on the (statistical) properties of the propagation channel. A discussion of the channel measurements is, therefore, given in Section 5.1.

### 5.1. Channel

If the channel cannot be measured at all possible transmitter and receiver locations in the environment, then the statistics of the multipath depend on the choice of these locations. Figure 7 shows the floor plan of the seminar room (cf. Section 4). The antenna location of the UWB transmitter ${\mathrm{Tx}}_{S}$ and of the interference source ${\mathrm{Tx}}_{I}$ are fixed for all results provided in this section. The green rectangle represents the area where the receiver was placed at 8000 evenly distributed locations.

**Figure 8:**
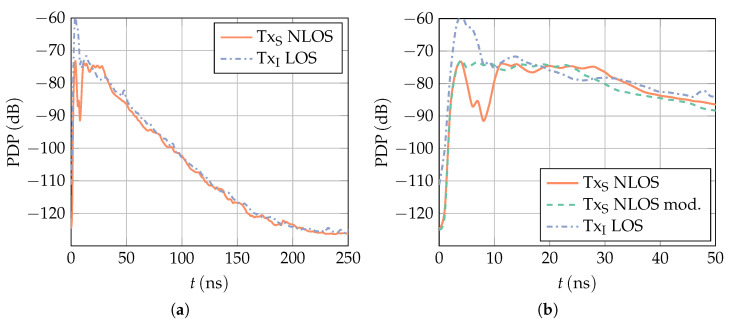
PDP obtained from 8000 measurements in a seminar room (cf. Section 4) aligned at the first path. The red line corresponds to the UWB channel (transmitter is placed at ${\mathrm{Tx}}_{S}$ in Figure 7) and the blue dash-dotted curve to the interference channel (transmitter is placed at ${\mathrm{Tx}}_{I}$), measured under NLOS and LOS conditions, respectively. The green dashed line corresponds to the modified UWB channel, where the multipath components are shifted towards the LoS component. (**b**) is a zoomed-in version of (**a**).

In ToA estimation, the channel is typically characterized by its PDP because it provides information about the mean power of multipath components at a certain delay. Figure 8 shows the PDP for the transmitter and receiver locations described above. It is obtained by averaging the squared absolute value of all measurements aligned at the first path. The UWB channel was measured under NLoS conditions (cf. Figure 6), and the interference channel under LoS conditions. It can be seen in Figure 8a that there is noticeable energy in the channel up to a delay of 200 ns before the curve flattens out. Figure 8b shows the same plot in greater detail for small delay values. Here it is evident that, in the UWB channel, there is a gap after the first path before it reaches a plateau introduced by the first strong multipath components. This gap corresponds to a distance of about 2 m between the first path and the first multipath components and is a result of the rather central placement of the transmitter and receiver within the room. As such a gap is typically not present in practical scenarios, the measured channel is modified in the post-processing. For every channel measurement, the first strong multipath component is identified and and all successive samples are shifted 1.8 m towards the first path by an overlap-and-add method. The PDP of the resulting channel is also shown in Figure 8b. Note that the same height of the first peak and the plateau in the NLoS case might be misleading at first sight. Due to alignment with regard to the first path, the power at this path is always summed up coherently, while the multipath components arrive at different delays and are averaged to a lower level. The first path is generally much weaker (about 10 dB on average) than the first multipath components. It is further apparent that the first peak in the interference channel has about twice the width of the peak in the UWB channel. This is caused by the placement of the interference transmitter ${\mathrm{Tx}}_{I}$ right next to a cabinet with a glass front. Almost all of the energy transmitted in the direction of the cabinet is reflected back and interferes constructively or destructively (depending on the receiver location) with the signal transmitted in the direction of the receiver, which results in a widened receive pulse.

### 5.2. Performance of the ToA Estimation

In order to evaluate the proposed method, propagation of the UWB signal and the interference signal over their respective channels is performed in a simulation using the measurement results described above. This enables the investigation with regard to a variety of parameters presented below. The pulse s(t) is a raised-cosine pulse (in compliance with ([7], Section 16)), with a roll-off factor of β=0.5 and a bandwidth of B=500 MHz. The interference signal is a Gaussian random process, effectively bandlimited to $B_{I}$ at a center frequency of 50 MHz below the UWB center frequency $f_{c}$. The virtual arrays are formed at 4000 positions within the measurement area in Figure 7. We evaluate the estimation performance in terms of the distance error given by

(33) $$\epsilon_{d}=c_{0}\epsilon_{\tau }\;,$$

where $\epsilon_{\tau }$ is given in Equation (32), and $c_{0}$ is the speed of light. The SINR is defined by the ratio of the peak power of the pulse $g^{\left(0\right)}$ at the index $m^{\left(0\right)}$ corresponding to the ToA and the mean power of the perturbation vector $p^{\left(0\right)}$

(34) $$\mathrm{SINR}=20\log_{10}\left(\frac{|g_{m^{\left(0\right)}}^{\left(0\right)}|}{\frac{1}{N}{∥p^{\left(0\right)}∥}_{}}\right)\;,$$

and the interference power is 30 dB above the noise power. Note that here, the mean is not with regard to multiple realizations of the random vector $p^{\left(0\right)}$, but with respect to the samples within one realization.

#### 5.2.1. LCMV Parameterization

**Figure 9:**
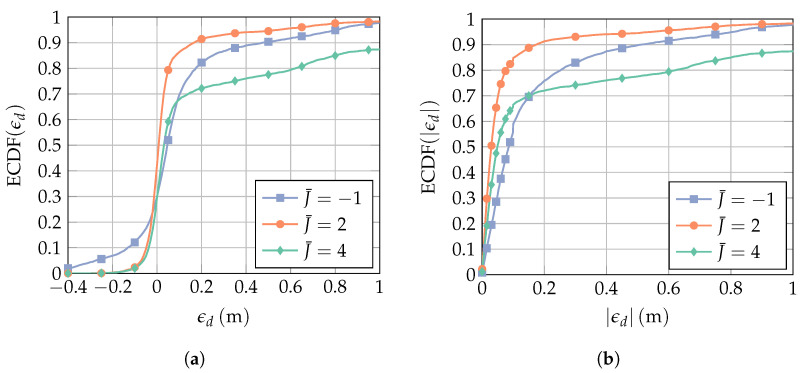
ECDF of (**a**) the ToA estimation error $\epsilon_{d}$ and (**b**) the absolute value of $\epsilon_{d}$ for various numbers of point constraints *J*. The receiver array is a 1×2 ULA with broadside in the *x*-direction (cf. Figure 7), and the filter length is L=4. The interference bandwidth is $B_{I}=160\;M\mathrm{Hz}$, the SINR is 9 dB, the TINR is set to 10 dB, and the AoA θ is assumed to be known. The legend is valid for both figures.

Let us first consider the lowest-dimensional case of a 1×2 receiver array, i.e., K=2 and a filter length of L=4. First, we evaluate the performance of the proposed method with regard to the number of point constraints *J*. Figure 9 shows the empirical cumulative distribution function (ECDF) of the distance error. The ECDF is normalized to the total number of observations. It does not, therefore, attain a probability of one if there are observations for which no valid estimate is obtained. The three curves show the results for different numbers of point constraints *J*. As the best choice of *J* is usually in the vicinity of KL/2, it is represented by the centered number of point constraints

(35) $$\bar{J}=J-KL/2\;.$$

It is evident in Figure 9a that most of the errors are centered around zero. These errors are introduced by correctly detecting the first path. The errors above 0.2 m arise from erroneously detecting multipath components. If the chosen $\bar{J}$ is too small ($\bar{J}=-1$), then the desired signal is distorted, and the local errors in the vicinity of the LoS increase. If the chosen $\bar{J}$ is too large ($\bar{J}=4$), then the desired signal form is well preserved but the interference mitigation capability decreases, and the estimation error becomes increasingly dominated by multipath errors.

**Figure 10:**
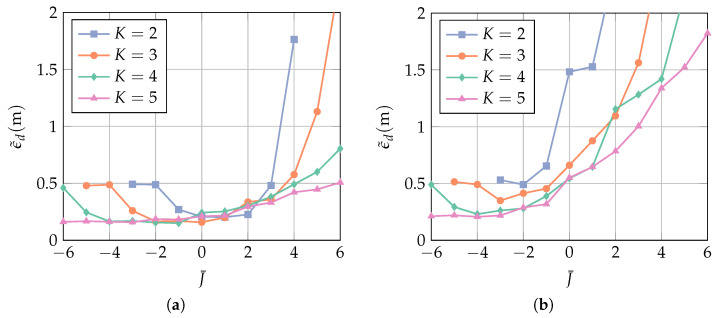
MAE over the number of constraints $\bar{J}$ for (**a**) SINR=9 dB and (**b**) SINR=0 dB. The receiver array is a ULA with broadside in the *y*-direction (cf. Figure 7). The number of constraints $\bar{J}$ is chosen to be the optimal value for each *L*. The interference bandwidth is $B_{I}=160\;M\mathrm{Hz}$, and the AoA θ is assumed to be known.

In order to quantify the performance of the ToA estimation, we define the mean absolute error (MAE) as

(36) $${\tilde{\epsilon }}_{d}=\frac{1}{N_{\mathrm{pos}}}\sum_{i=1}^{N_{\mathrm{pos}}}\left|\epsilon_{d,i}\right|\;,$$

where $\epsilon_{d,i}$ is the distance error at the *i*th position, and $N_{\mathrm{pos}}$ is the total number of positions. Figure 10 shows the MAE over a range of values of $\bar{J}$ for different numbers of antenna elements *K*, where the array configuration is a uniform linear array (ula). The U shape of the curves follows the same explanation as for Figure 9: if $\bar{J}$ is too small, then the desired pulse is distorted, and the MAE is increased due to local errors. The MAE for the two smallest possible values of $\bar{J}$ is equal because one constraint leads to a similar signal distortion as two constraints. If $\bar{J}$ is too large, then the degrees of freedom are too low for a sufficient interference suppression, and the MAE is dominated by multipath errors.

For SINR=9 dB, the optimal values for $\bar{J}$ are larger than for SINR=0 dB. This is explained as follows. When the SINR is large, the LoS is detected correctly without a large amount of interference suppression. In this case, the MAE is dominated by local errors, which are small for a large number of constraints $\bar{J}$. When the SINR is low, a high amount of interference suppression is needed in order to minimize multipath errors. This is achieved with more degrees of freedom, i.e., when $\bar{J}$ is low. The MAE at the right side of the minimum is much larger than on the left side because multipath errors are typically much larger than local errors (cf. Figure 3). In addition, for any fixed $\bar{J}$, using more antenna elements K decreases the MAE, as expected, due to having more spatial information available.

**Figure 11:**
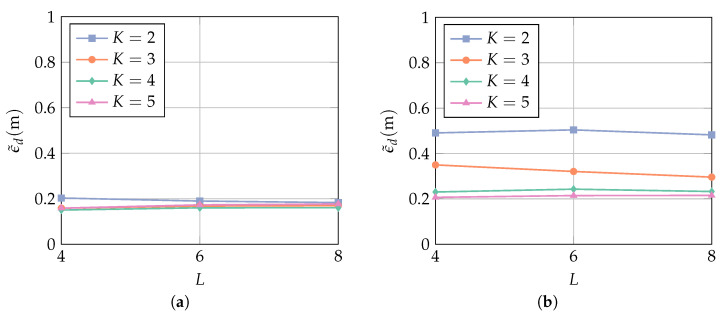
MAE over the filter length *L* for (**a**) SINR=9 dB and (**b**) SINR=0 dB. The receiver array is a ula with broadside in the *y*-direction (cf. Figure 7), and the filter length is L=4. The interference bandwidth is $B_{I}=160\;M\mathrm{Hz}$, and the AoA θ is assumed to be known.

In Figure 11, the MAE is evaluated with regard to the number of antenna elements *K* and the filter length *L*. It is evident that no performance improvement can be gained by investing in a larger filter length. In order to minimize the computational complexity, the lowest value of *L* is most favorable. While, for SINR=9 dB, all curves approach the same MAE, additional antenna elements improve the performance significantly at SINR=9 dB. It is seen, in particular, that for K=4 and K=5, the MAE is at almost the same value for an SINR difference of 9 dB. Note, however, that a larger *K* results in a higher computational complexity.

The number of constraints $\bar{J}$ in Figure 11 is chosen to be optimal for each *K* and for both values of the SINR. This optimum corresponds to the location of the minima in Figure 10, but the SINR is unknown a priori. While the interference-plus-noise power is given by the diagonal elements of ${\hat{R}}_{p_{m}p_{m}}$ in Equation (26), the signal power depends on the ToA of the first path $\tau^{\left(0\right)}$, which is the quantity to be estimated. In a scenario where multiple successive measurements are performed, one approach is to start with a small value for $\bar{J}$ in order to increase the probability of correctly detecting the first path. Once the ToA $\tau^{\left(0\right)}$ has been estimated, the power of the first path can be estimated. This value can then be used to determine $\bar{J}$ for the next measurement. In practice, this is a reasonable approach if the duration between two successive measurements is not too large, i.e., when the first path power is not likely to vary strongly between the measurements.

#### 5.2.2. AoA and SINR Uncertainty

**Figure 12:**
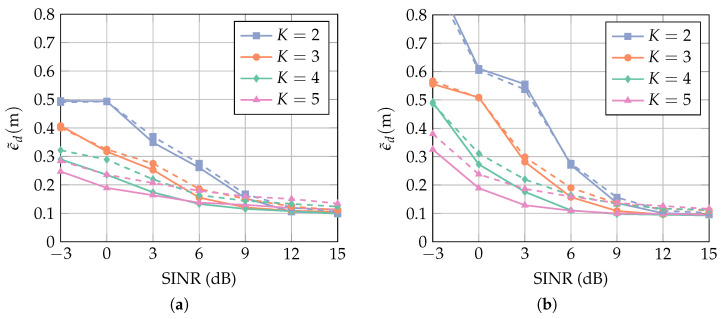
MAE over SIR for interference bandwidth (**a**) $B_{I}=160\;M\mathrm{Hz}$ and (**b**) $B_{I}=320\;M\mathrm{Hz}$. The curves show the mean value of the results for the cases in which the receiver array is a ULA with broadside in the *x*-direction and in the *y*-direction (cf. Figure 7). Solid lines show the results for a known AoA θ, and dashed lines for $\sigma_{\theta }=0.3\mathrm{rad}$. The number of constraints $\bar{J}$ is optimal for each *K* and SINR.

The AoA θ of the desired signal enters the model via the constraint matrix C. If θ is unknown a priori, then it has to be estimated, and the estimation is subject to errors. When C is calculated for a value of θ that deviates from the true value, the desired signal is distorted, which results in larger local errors. A method to mitigate this effect without increasing the computational complexity is described in Section 3.4. In order to evaluate the sensitivity of the proposed method to uncertainties of θ, we assume that θ follows a wrapped normal distribution

(37) $$\theta ∼\mathrm{WN}(\mu_{\theta },\sigma_{\theta })\;,$$

where the mean $\mu_{\theta }$ is the true value at each location and $\sigma_{\theta }$ is the standard deviation in radian. Figure 12 shows the MAE over the SINR for $\sigma_{\theta }=0$ (known θ) and $\sigma_{\theta }=0.3\mathrm{rad}$. For K=2 and K=3, the curves almost coincide, because for a low number of antenna elements, the array response does not change rapidly in the spatial domain. For K=4 and K=5, the array response is more sensitive in the spatial domain and, consequently, the constraints are not strictly satisfied for an AoA which deviates from the true value. This results in a slightly larger MAE. The difference, however, is in the sub- 10 cm regime, which shows that the method is robust to a certain amount of deviations in θ. Comparing Figure 12a and Figure 12b, it is seen that this behavior is independent of the interference bandwidth $B_{I}$.

**Figure 13:**
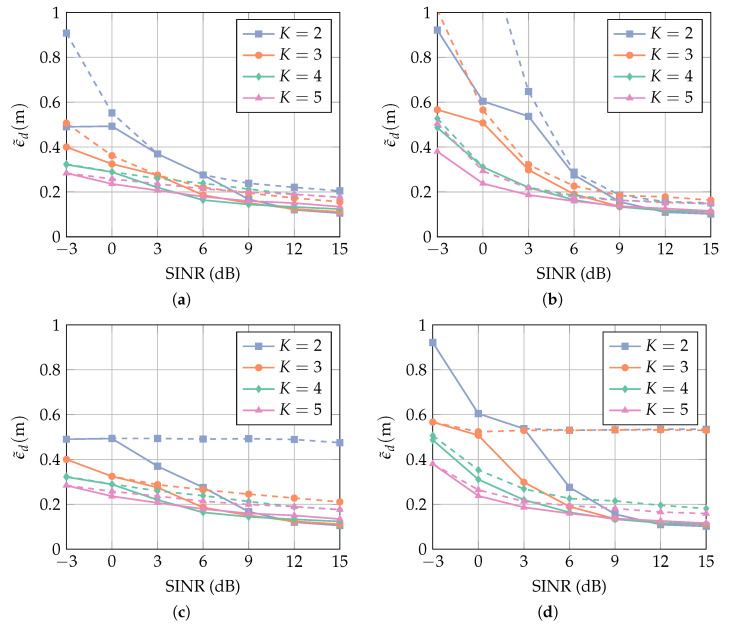
MAE over SIR for interference bandwidths (**a**,**c**) $B_{I}=160\;M\mathrm{Hz}$ and (**b**,**d**) $B_{I}=320\;M\mathrm{Hz}$. The curves show the mean value of the results for the cases in which the receiver array is a ULA with broadside in the *x*-direction and in the *y*-direction (cf. Figure 7). Solid lines show the results when $\bar{J}$ is optimal for each *K* and SINR. Dashed lines correspond to a fixed $\bar{J}$ given by (**a**,**b**) $\bar{J}=-1$ for K=2, $\bar{J}=-3$ for K=3, $\bar{J}=-4$ for K=4, $\bar{J}=-4$ for K=5, and (**c**,**d**) $\bar{J}=-2$ for K=2, $\bar{J}=-4$ for K=3, $\bar{J}=-5$ for K=4, $\bar{J}=-5$ for K=5. The standard deviation of θ is $\sigma_{\theta }=0.3\;\mathrm{rad}$.

It is shown in Section 5.2.1 that the optimal value of $\bar{J}$ depends on the SINR, and that the SINR is unknown in general. Figure 13 compares the MAE for a fixed value of $\bar{J}$ and an adaptively chosen optimal value for $\bar{J}$. The optimal value is determined by the minimum of the curves in Figure 10 for each value of *K* and SINR. In Figure 13a,b, the chosen fixed $\bar{J}$ is rather high. This choice favors an undistorted signal over a high interference suppression. For high SINR values, the error is therefore not much larger than in the optimal case. In the low SINR regime, the poor interference suppression capability results in higher multipath errors, and the MAE is significantly larger than in the optimal case. In Figure 13c,d, the chosen fixed $\bar{J}$ is rather low, and the behavior described above is reversed. The error floor in this case is at roughly 0.5 m due to the pulse distortion. At low SINR values, the fixed value for $\bar{J}$ coincides with the optimal value and, consequently, the MAE also coincides with the optimum. Note that, for a fixed value of $\bar{J}$, no knowledge about the SINR is required. If multiple successive measurements are available, a rough estimate of the SINR can be obtained as described in Section 5.2.2, and $\bar{J}$ can be determined adaptively.

#### 5.2.3. Comparison to Interference Blocking

**Figure 14:**
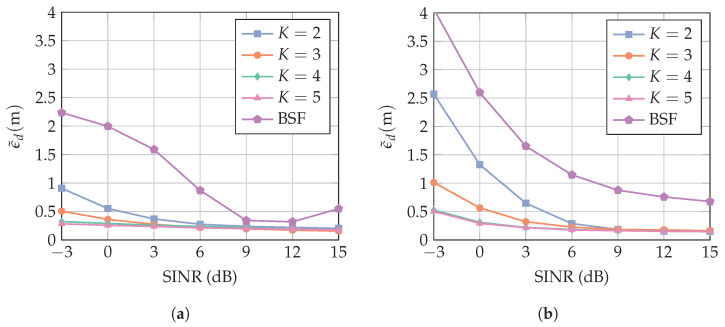
MAE over SIR for (**a**) interference bandwidths $B_{I}=160\;M\mathrm{Hz}$ and (**b**) $B_{I}=320\;M\mathrm{Hz}$. The curves show the mean value of the results for the cases in which the receiver array is a ULA with broadside in the *x*-direction and in the *y*-direction (cf. Figure 7). The number of constraints is fixed (cf. Figure 13), and the standard deviation of θ is $\sigma_{\theta }=0.3\;\mathrm{rad}$.

Finally, we compare the proposed method to an approach where the interference mitigation is performed by band-stop filtering (BSF) the received signal. Figure 14 compares the results obtained for the two methods for different interference bandwidths $B_{I}$. The SINR is assumed to be unknown, and thus, the number of constraints $\bar{J}$ is fixed, as explained in Section 5.2.2. At $B_{I}=160\;M\mathrm{Hz}$, the proposed method performs significantly better in the low-SINR regime. With two antenna elements, the MAE is improved by more than 1 m compared to the BSF approach. The BSF introduces a deterministic smaller peak prior to the peak that corresponds to the LoS. For high SINR values, the detection threshold decreases in relation to the power of the first peak (cf. Figure 3), and the smaller peak prior to the LoS is detected erroneously. This explains the rise of the MAE at SINR=15 dB for the BSF. This effect could be equalized by adapting the detection threshold with regard to the SINR, but it was assumed before that the SINR is unknown. If the detection threshold is chosen to be larger, this effect would be shifted to higher SINR values. However, the MAE in the low-SINR regime would increase as well. At $B_{I}=320\;M\mathrm{Hz}$, the proposed method performs significantly better over the entire SINR range. The large error floor of the BSF is explained by the severe bandwidth reduction of the desired signal. The interference band with $B_{I}=320\;M\mathrm{Hz}$ corresponds to 64% of the signal bandwidth *B*. Consequently, a large fraction of the desired signal is suppressed by the BSF. This results in a merge of several multipath components, and thus, in a shift of the first peak towards higher delay values.

#### 5.2.4. Complexity

The complexity of the proposed method is determined by the estimation of $R_{p_{m}p_{m}}$ in Equation (26), the inversion of the matrices $R_{p_{m}p_{m}}$ and $C^{H}R_{p_{m}p_{m}}C$, and the matrix multiplications in Equation (21). In the case of K=2, L=4, and J=3 ($\bar{J}=-1$), the perturbation covariance matrix $R_{p_{m}p_{m}}$ is 8×8-dimensional, the constraint matrix C is 8×3-dimensional, and $C^{H}R_{p_{m}p_{m}}C$ is 3×3-dimensional. The number of arithmetic operations required for the inversion of an n×n-dimensional matrix is approximately $2n^{3}/3$ ( the exact number depends on the used algorithm). The inversion of $R_{p_{m}p_{m}}$ and $C^{H}R_{p_{m}p_{m}}C$, therefore, requires approximately 340 and 18 operations, respectively. For the estimation of $R_{p_{m}p_{m}}$, we used 10 sample vectors, which results in 8×8×10=640 operations. The multiplication of a k×l-dimensional by an l×m-dimensional matrix requires (l+l−1)km operations. All of the matrix multiplications in Equation (21) sum up to 975 operations. This results in a total number of approximately 2000 operations for the calculation of the optimal filter coefficients in Equation (21). A low-dimensional implementation of the proposed method is, therefore, indeed of low complexity.

## 6. Conclusions

We proposed a low-complexity approach to wideband interference mitigation and ToA estimation in an UWB system. The method combines an LCMV processor with a detection-based ToA estimator.

In order to evaluate the performance of the proposed method with regard to the MAE, we conducted a large number of virtual array channel measurements for both the UWB and the interference channel.

The most important parameters involved in the LCMV processing are the number of antenna elements, the filter length per antenna element, and the number of constraints. While the filter length has almost no impact on the MAE, additional antenna elements lead to a significant improvement in low-SINR regimes. The optimal number of constraints depends on the SINR, which is unknown in general. Deviations from this optimum are critical only for a low number of antenna elements at low SINR values. Using a probabilistic model for the constraint matrix, the method is also robust to uncertainties in the AoA. Furthermore, we compared the results to those obtained by using a band-stop filter approach. The LCMV approach performs significantly better over the entire SINR range, especially when the interference bandwidth exceeds 60% of the signal bandwidth.

Based on the presented results, we conclude that the proposed method is suitable when the interference bandwidth occupies a large fraction of the signal bandwidth and when computational power is limited.

## Data Availability

Not applicable.

## Abbreviations

The following abbreviations are used in this manuscript:

AoA	angle of arrival
BSF	band-stop filter
CMP	conical monopole antenna
DM	dense multipath
DMC	dense multipath component
ECDF	empirical cumulative distribution function
HRP PHY	high-repetition pulse physical layer
LCMV	linear constraint minimum variance
LoS	line of sight
MAE	mean absolute error
MF	matched filter
ML	maximum likelihood
NLoS	non-line of sight
pdf	probability density function
PDP	power delay profile
SINR	signal-to-interference-plus-noise ratio
ToA	time of arrival
TINR	threshold-to-interference-plus-noise ratio
ULA	uniform linear array
UWB	ultra-wideband
VNA	vector network analyzer
WSS	wide-sense stationary
